# The draft genome of *Ditylenchus dipsaci*

**DOI:** 10.21307/jofnem-2019-027

**Published:** 2019-05-27

**Authors:** Benjamin Mimee, Etienne Lord, Pierre-Yves Véronneau, Rick Masonbrink, Qing Yu, Sebastian Eves-van den Akker

**Affiliations:** 1 Agriculture and Agri-Food Canada, St-Jean-sur-Richelieu Research and Development Centre, St-Jean-sur-Richelieu, J3B 3E6 QC, Canada; 2 Iowa State University, Ames, IA; 3 Agriculture and Agri-Food Canada, Ottawa Research and Development Centre, K1A 0C6 Ottawa, ON, Canada; 4 Department of Plant Sciences, University of Cambridge, Cambridge

**Keywords:** Canu, Genome assembly, PacBio, Stem and bulb nematode

## Abstract

*Ditylenchus dipsaci* is a devastating pest to many crops worldwide. We present the first genome sequence for this species, produced with PacBio sequencing and assembled with CANU.

The stem and bulb nematode, *Ditylenchus dipsaci*, is a plant-parasitic nematode affecting over 500 plant species with major destruction occurring in onion, garlic, beet, strawberry, broad bean, potato, alfalfa, and oat (Sturhan and Brzeski, 1991). It is a quarantine pest in Europe (EPPO, 2004) and well known in garlic production where it can cause losses of up to 90% (Abawi and Moktan, 2010). The species exhibits a variety of host preferences for which biological groups were proposed (Sturhan and Brzeski, 1991). In this study, we report the first genome assembly of *D. dipsaci*, a resource that will improve our understanding of this important parasite.

A population of *D. dipsaci* (E-105) was obtained from the *Canadian National Collection of Insects, Arachnids, and Nematodes* and originally isolated from garlic in Northern Ontario (Qiao et al., 2013). The nematodes were multiplied *in vitro* on pea sprout inoculated with only 15 mixed-stages individuals, as described in Poirier et al. (2019), to reduce heterozygosity. DNA was extracted from ~7,500 mixed-stage *D. dipsaci,* using the Qiagen DNeasy Blood & Tissue Kit (Valencia, CA). *MUGQIC* (Montréal, Canada) performed the library preparation (sheared large insert) and sequencing on a PacBio RSII instrument using 12 SMRT cells yielding 941,946 long reads at a mean size of 10,207 bp for an estimated coverage of 42×. Two assemblies were generated using CANU v1.7 (Koren et al., 2017), one with default parameters and one optimized for high heterogeneity (corMhapSensitvity = high, corMinCoverage = 0, and correctedErrorRate = 0.105). These pre-assemblies were merged using Quickmerge (Chakraborty et al., 2016) and decontaminated using Blobtools v1.0 (Laetsch and Blaxter, 2017). Before scaffolding, the assembly contained 3,559 contigs with a N50 of 152 kb. Contigs were then scaffolded using MeDuSa v1.6 (Bosi et al., 2015) with the genomes of *Caenorhabditis elegans* (GCA_000002985.3; The C. elegans Sequencing Consortium, 1998), *D. destructor* (GCA_001579705.1; Zheng et al., 2016), *Globodera pallida* (GCA_000724045.1; Cotton et al., 2014), *G. rostochiensis* (GCA_900079975.1; Eves-van den Akker et al., 2016), and *Meloidogyne incognita* (GCA_900182535.1; Abad et al., 2008). Scaffolding was further improved using Rascaf (Song et al., 2016) and 13,555,476 PE RNA-seq reads (Phred score > 20), obtained from mixed-stage *D. dipsaci* (Illumina HiSeq 2 × 100 bp, TruSeq Library Prep Kit). Then, Purge Haplotigs (Roach et al., 2018) resolved highly polymorphic regions and reduced artifactual duplication.

The *D. dipsaci* genome assembly is comprised of 1,394 scaffolds, a largest scaffold of ~3.6 Mb, 22 scaffolds >1 Mb, and a N50 of 287 kb. The assembly is 227,234,012 bp (227.2 Mb), double the size of the *D. destructor* assembly (111.1 Mb). We used flow cytometry on *D. dipsaci* nuclei to investigate this discrepancy, revealing a haploid genome size of 160 to 170 Mb. Thus, obtaining 227 Mb with heterozygous material is not surprising. The GC content of the *D. dipsaci* assembly was 37.5% which is comparable to *D. destructor* (36.8%), *C. elegans* (35.4%), *G. rostochiensis* (38.1%), and *G. pallida* (36.7%). A completeness assessment with BUSCO v 3.0.2 (Simão et al., 2015) using the Nematoda_odb9 database revealed 537 complete sequences (506 single-copy, 31 duplicated), 133 fragmented, and 312 missing on a total of 982 BUSCO genes searched in *D. dipsaci.* The number of complete genes was higher than *G. pallida* (431) but lower than *D. destructor* (749). Gene prediction was performed with Braker 2.1.0 (Hoff et al., 2015) on a genome masked with Repeatmodeler/Repeatmasker 4.0.7 (Chen, 2004), leaving simple repeats unmasked. Transcript information was relayed to Braker via HISAT2 2.1.0 alignments of *D. dipsaci* RNA-seq and exonerate 2.4.0 aligned *Ditylenchus destructor* proteins to produce a gene annotation of 26,428 putative genes. On average, these genes contained seven exons (max = 114) with a mean length of 120 bp (from 2 to 9,194 bp) and six introns (max = 113) with a mean length of 515 bp (from 36 to 27,260 bp).

*GenBank accession numbers*: The raw sequences and genome assembly files are available under the NCBI BioProject PRJNA498219.
